# Sustained Virological Response on Second-Line Antiretroviral Therapy following Virological Failure in HIV-Infected Patients in Rural South Africa

**DOI:** 10.1371/journal.pone.0058526

**Published:** 2013-03-11

**Authors:** Annelot F. Schoffelen, Annemarie M. J. Wensing, Hugo A. Tempelman, Sibyl P. M. Geelen, Andy I. M. Hoepelman, Roos E. Barth

**Affiliations:** 1 Department of Internal Medicine and Infectious Diseases, University Medical Centre Utrecht, Utrecht, The Netherlands; 2 Department of Virology, University Medical Centre Utrecht, Utrecht, The Netherlands; 3 Ndlovu Care Group, Elandsdoorn, Limpopo, South Africa; 4 Department of Paediatric Infectious Diseases, University Medical Centre Utrecht, Utrecht, The Netherlands; University of Pittsburgh, United States of America

## Abstract

**Objective:**

This study aims to describe the virological, immunological and clinical efficacy of protease inhibitor (PI)-based second-line antiretroviral therapy (ART) in rural South Africa.

**Methods:**

An observational cohort study was performed on 210 patients (including 39 children) who initiated PI-based second-line therapy at least 12 months prior to data collection. Biannual clinical, immunological and virological monitoring was performed. Primary endpoints were adequate virological response (plasma HIV-1 RNA<400 copies/ml), full virological suppression (plasma HIV-1 RNA<50 copies/ml) and treatment failure (virological failure (plasma HIV-1 RNA>1000 after initial virological response) or on-going viremia (plasma HIV-1 RNA never<400 copies/ml for more than six months)). Data were analyzed by an on-treatment (OT) and intention-to-treat (ITT) approach. Analyses were primarily performed on the group of patients who switched following first-line virological failure.

**Results:**

Median duration of follow-up after switch to second-line treatment was 20 months [IQR 11–35]. 191 patients had switched to second-line ART due to first-line virological failure. 139/191 of them (72.8%, ITT) were in care and on treatment at the end of follow-up and 11/191 (5.8%, ITT) had died. After twelve months, an adequate virological response was seen in 92/128 patients (71.9%, OT), of which 78/128 (60.9%, OT) experienced full virological suppression. Virological response remained stable after 24 months. Virological efficacy was similar amongst adult and pediatric patients. As in first-line ART, we observed a lack of correlation between virological failure and WHO-defined immunological failure.

**Conclusions:**

Good virological outcomes following first-line failure can be achieved with PI-based, second-line antiretroviral therapy in both adult and pediatric patients in rural South Africa. Retention rates were high and virological outcomes were sustainable during the two-year follow-up period, although persisting low-level viremia occurred in a subset of patients. The observed viro-immunological dissociation emphasizes the need for virological monitoring.

## Introduction

Most of the people who are HIV-infected globally, reside in sub-Saharan Africa. The massive roll-out of antiretroviral therapy (ART) that has taken place in this region since 2003, resulted in a stark increase in the number of HIV-infected patients receiving treatment. Despite these impressive achievements, treating HIV-infected people in resource-limited settings (RLS) remains challenging.

The World Health Organization (WHO) recommends combining two nucleoside reverse transcriptase inhibitors (NRTIs) and one non-nucleoside reverse transcriptase inhibitor (NNRTI) as first-line ART in RLS. [1] The efficacy of these regimens has been studied extensively. In patients surviving and remaining in care after the initial months of treatment, improvements in clinical and immunological outcome measurements were comparable to those observed in Western countries. [2] Unfortunately, early outcomes are generally worse, as a result of high mortality rates soon after treatment start. [3], [4] Studies performed in RLS are typically set in urban areas. However, data from non-urban settings are limited. Previously, we analyzed the efficacy of first-line ART in a cohort of HIV-infected patients in a rural setting in South Africa. [5] Virological treatment failure was seen in twenty percent of patients who survived the first three months of therapy, which is comparable to other reports on the efficacy of first-line ART in RLS. [2], [6], [7]

Second-line antiretroviral therapy in RLS generally consists of a ritonavir-boosted protease inhibitor (PI), mostly lopinavir, combined with a dual NRTI backbone. [1] As an HIV infection requires life-long treatment, and as there will always be a proportion of patients that experiences virological failure during therapy, the need for second- and consecutive lines of ART regimens will increase over time, even when first-line efficacy is generally good.

The efficacy of second-line ART programs in RLS may be different from that previously described for western countries for several reasons. First, HIV subtypes circulating in African countries vary, with subtype C being most frequent, whereas subtype B is most prevalent in western countries. [8] Different HIV subtypes may respond differently to the various antiretroviral agents and may use other pathways for selecting resistance mutations. It is not clear if this concerns reverse transcriptase mutations only or those in protease as well. [2], [9] Second, in western countries a second-line ART regimen is commonly composed following genotypic resistance testing which enables the selection of a regimen with an optimal genetic barrier. In RLS genotypic resistance testing on an individual basis is generally not feasible and second-line regimens are therefore mostly initiated empirically. In practice, this usually means a switch of the NNRTI to a boosted PI. The NRTI backbone is partly retained, with the continuation of lamivudine and, depending on the availability of alternative options, one new NRTI. Due to NRTI cross-resistance, this approach frequently results in the use of second-line regimens with a suboptimal backbone, potentially affecting treatment efficacy. This may be of limited clinical relevance, as PI-monotherapy generally results in adequate virological outcomes in a majority of patients. [10] Still, the actual efficacy of such a strategy remains to be seen.

Data on the efficacy of second-line ART in RLS are rare; the few existing studies are limited by cohort size and follow-up duration and are mainly based in urban areas. Early reports generally show good short term outcomes with high rates of survival, immune reconstitution and virological suppression on second-line therapy. [11]–[13] Reported virological failure rates are varying. A recent review and earlier studies describe relatively high proportions of patients experiencing virological failure, mostly within the first six months after switch to a second-line regimen. [14]–[17] Longer term follow-up data on second-line regimens are sparse, due to the relatively short time period that such regimens have been in use and to the limited availability of virological monitoring in these regions. Our study describes long-term virological, as well as immunological and clinical, efficacy of PI-based second-line ART in a rural setting using regular plasma HIV-1 viral load monitoring.

## Methods

### Setting/study site

Elandsdoorn is a township situated in a poor, rural area in Limpopo, a province in the northeast of the Republic of South Africa. HIV prevalence among antenatal clinic attendees in 2010 was 21.9% in this province. [18] Ndlovu Medical Centre is a non-governmental organization in Elandsdoorn (www.ndlovucaregroup.co.za) that provides “paid for service” primary health care, as well as prevention-, tuberculosis- and HIV/AIDS-programs which are donor-funded and free of charge for the patient. It serves a population of 120,000–140,000 people and around 4,000 HIV-infected patients are in care at the clinic. Patients are seen by doctors and HIV-counselors during each clinic visit in order to provide information and to stimulate treatment adherence. In a previous study, genotyping in this cohort showed that all viruses were of HIV-1 subtype-C origin. [19]

### Ethics statement

Medical ethics review was not required for this observational cohort study that was a follow-up of previous studies conducted on this cohort. [5], [19]–[21] Data were extracted from medical charts retrospectively. All information and results had been collected previously in the course of routine clinical care in the treatment evaluation program. Privacy of patients was provided by analyzing data anonymously. All patients signed informed consent prior to the start of ART for data collection and evaluation.

### Patients and monitoring

For this study, all patients who initiated (standard public-sector) PI-based second-line therapy at the Ndlovu Medical Centre at least 12 months prior to data extraction (between March 2004 and October 2010) were included. All patients had been previously treated with a WHO-recommended, NNRTI-based first-line ART regimen. Tenofovir was not prescribed for children, as current guidelines do not encourage its use in prepuberty. For second-line regimens, the NNRTI was replaced by ritonavir-boosted lopinavir in all patients, and combined with various NRTI-backbones. Pediatric dosages of lopinavir/ritonavir were supplied as oral solution, while for adults, the capsule formulation (which needs to be refrigerated) was replaced by tablets in 2008.

Virological failure on first-line treatment was the main reason to switch to second-line therapy. In certain cases, there were other reasons to switch, such as adverse drug events while using first-line treatment. Virological failure on first-line ART was defined as a plasma HIV-1 viral load rising to over 1000 copies/ml after initial virological suppression (plasma HIV-1 viral load<400 copies/ml), or continued detectable plasma HIV-1 viral loads over 400 copies/ml, after at least six months of ART.

Plasma HIV-1 viral loads (System 340 bDNA analyzer, Bayer AG, Leverkusen, Germany) and CD4 counts (FACSCalibur system, Becton Dickinson Biosciences, San Jose, CA) during treatment were measured biannually in the laboratory on site. Shortly before and three months after switch to second-line therapy, additional plasma HIV-1 viral load measurements were performed.

### Data extraction

Data were retrospectively collected from the medical charts, using routinely collected follow-up data. Baseline information regarding previous first-line therapy, social status, age and gender were collected from the charts, as was the duration of first-line therapy. Initial first-line regimens were described. A drug switch within the NRTI class because of adverse drug events was not considered to be a switch to second-line treatment. A change to at least one new antiretroviral drug class was considered to define a switch to second-line treatment.

### Statistical analysis

The primary endpoints were achieving virological success (either through full virological suppression or an adequate virological response), both at fixed time points after start of second-line treatment and at end of follow-up, and experiencing treatment failure during second-line therapy. Full virological suppression was defined as a plasma HIV-1 RNA<50 copies/ml and the definition of an adequate virological response was a plasma HIV-1 viral load<400 copies/ml. A plasma HIV-1 viral load>1000 copies/ml confirmed by a subsequent measurement after an initial adequate viral response was used to define virological failure. However, if no subsequent measurements were available, and the last plasma HIV-1 RNA was above 1000 copies/ml, patients were also considered to experience virological failure. The continued (more than six months) detection of a plasma HIV-1 RNA>400 copies/ml was considered to represent on-going viremia. Both virological failure and on-going viremia were regarded as second-line treatment failure. Virological responses were determined both for the entire group, and separately for adults and children (age<15 years).

Secondary endpoints were both clinical parameters (mortality from all causes, being lost to follow-up or being transferred out) and immunological parameters (absolute change in CD4 counts). A patient was considered lost to follow-up if he or she had not shown up at the clinic for six months or longer before the moment of data extraction and if there was no available information on death or transfer out to another clinic. Immunological responses were only analyzed for the adult patients and children of 5 years and older, as CD4 percentages, the preferred immunological parameter for HIV-infected children younger than five, were not available. Immunological failure for adult patients was defined according to the WHO guidelines as either a CD4 count after six months of therapy below 100 cells/mm^3^ or below the pre-therapy count, or a 50% decline from the on-treatment peak CD4 count value. [1]

All analyses on second-line ART outcome were primarily performed on the group of patients who had switched following first-line virological failure and were repeated for the total group of patients.

Data were analyzed by an intention-to-treat (ITT) and on an on-treatment (OT) approach at fixed time points, for which a window period of three months prior to and following that time point was accepted for data collection. The end of follow-up for virological or immunological response was defined as the most recent date with available results on either plasma HIV-1 RNA or CD4 cell count, respectively, before the time of data extraction, death, transfer out or being lost to follow-up. Continuous data were compared with the Student's t-test or the paired t-test as appropriate. Proportions were compared with the chi-square-test and data that were not normally distributed were analyzed via the Mann-Whitney U or Wilcoxon rank test. Kaplan-Meier survival analyses were used to estimate the time from switch to second-line ART to full virological suppression. To define predictors of treatment failure, univariate analyses were performed with the following determinants: age, gender, being a child, first-line ART regimen (zidovudine versus stavudine as NRTIs and nevirapine versus efavirenz as NNRTIs), change of CD4 count during first-line treatment, CD4 count at the moment of switch to second-line ART, calendar year of switch, duration of first-line treatment, duration of documented first-line treatment failure, and duration of second-line treatment. A multivariable analysis was performed by using the logistic regression model, including all variables that were associated with the outcome (*P*<0.50) in univariate analysis. A *P*-value ≤0.05 was considered statistically significant. Data were processed and statistical analyses were done using SPSS version 17.0.

## Results

### Baseline Characteristics

A total of 210 patients were started on a PI-based, second-line antiretroviral therapy at least 12 months prior to data collection, and were included in analyses. In 191/210 patients (91.0%) the reason to switch to second-line therapy was first-line treatment failure. The other 19 patients (9.0%) mainly switched for reasons of toxicity. Only eight of those had an undetectable plasma HIV-1 viral load at the moment of switch.

Most patients were female (69.0% (145/210)) and 18.6% (39/210) of patients were children under 15 years. 27 of these children were 5 years or older at the time of switch. Unemployment amongst adults was high (68.4% (117/171)).

Patients were severely immune compromised at initiation of first-line ART, as shown by the low median CD4 counts (62 cells/mm^3^ [IQR 18–139]) at treatment start. First-line ART consisted of lamivudine (3TC) with stavudine (d4T) or zidovudine (AZT) as NRTIs and efavirenz (EFV) (96/210 (45.7%)) or nevirapine (NVP) (112/210 (53.3%)) as NNRTI. The median duration of first-line treatment was 19 months [IQR 11–31 months]. The median duration between first documented virological failure on first-line ART and the moment of switch to second-line treatment was 6 months [IQR 3–11 months].

The median log plasma HIV-1 viral load in the total group at time of switch to second-line therapy (4.01 [IQR 3.40–4.48]) was significantly lower than that at time of ART initiation (4.94 [IQR 4.44–5.35]) (*P*<0.05). During the first-line treatment period, stavudine had often been replaced by zidovudine or another NRTI, decreasing stavudine usage from 69.5% in first- to 4.8% of patients in second-line regimens. In contrast, tenofovir was prescribed to only 0.6% (1/171) of adult patients during their first-line regimen, as compared to 19.3% (33/171) for second-line ART. The median duration of follow-up after switch was 20 months [IQR 11–35 months].

Baseline characteristics were similar for the entire group and the group of patients who had switched due to first-line treatment failure (data not shown). Baseline characteristics at start of first- and second-line treatment are summarized in Table 1.

**Table 1:** Baseline characteristics.

		N = 210
Male; N (%)		65 (31.0)
Median age in years [IQR]		33 [24–40]
Child (<15 years); N (%)		39 (18.6)
Unemployed adults (N = 171); N (%)		117 (68.4)
CD4 at start 1^st^ line ART (cells/mm^3^); median [IQR}; Adults		62 [18–139]
CD4 change from start 1^st^ line ART to switch (cells/mm^3^); median [IQR]; Adults		108 [43–201]
CD4 at time of switch (cells/mm^3^); median [IQR]; Adults		187 [93–299]
CD4 at time of switch (cells/mm^3^); median [IQR]; Children≥5 years		485 [308–983]
Log plasma HIV-1 viral load at start of 1^st^ line ART (copies/ml); median [IQR]		4.94 [4.44–5.35]
Log plasma HIV-1 viral load at time of switch (copies/ml); median [IQR]		4.01 [3.40–4.48]
Duration first line (months); median [IQR]		19 [11]–[31]
Documented duration virological failure before switch to second line regimen (months); median [IQR]		6 [3]–[11]
Duration follow-up second line (months); median [IQR]		20 [11–35]
**Initial first line regimen; N (%)**		
**NRTI**	lamivudine+stavudine	146 (69.5)
	lamivudine+zidovudine	61 (29.0)
	lamivudine+tenofovir	1 (0.5)
	unknown	2 (1.0)
**NNRTI**	nevirapin	112 (53.3)
	efavirenz	96 (45.7)
	unknown	2 (1.0)
**Second line regimen; N (%)**		
lamivudine+zidovudine+lopinavir/ritonavir		151 (71.9)
lamivudine+tenofovir+lopinavir/ritonavir		28 (13.3)
lamivudine+stavudine+lopinavir/ritonavir		10 (4.8)
emtricitabine+tenofovir+lopinavir/ritonavir		2 (1.0)
lamivudine+abacavir+lopinavir/ritonavir		1 (0.5)
other backbone+lopinavir/ritonavir		18 (8.6)

N: number of patients, IQR: interquartile range, NRTI: nucleoside reverse transcriptase inhibitor, NNRTI: non-nucleoside reverse transcriptase inhibitor.

### Patient retention and immunological response on 2^nd^ line ART

Retention rate at the end of follow-up (whilst on second-line ART) was 72.8%, with a total of 139/191 patients still being in care and on treatment among the 191 patients who had switched to second-line ART following first-line virological failure. 7.9% (15/191) of them were known to be transferred out to other clinics. Approximately one eighth of the cohort (16/191, 13.6%) was lost to follow-up at the moment of data extraction. 5.8% (11/191) of patients were known to have died during the observational period; half of them (6/11 (54.5%)) died within the first year after the switch to second-line ART.

Among these patients who had switched due to first-line failure, a cross-sectional on-treatment analysis at 12 months after start of second-line therapy showed a median CD4 count of 354 cells/mm^3^ [IQR 194–508] and 641 cells/mm^3^ [IQR 459–1245], based on 91 adult and 21 pediatric patients with data available at that time point, respectively. Among adults, the CD4 count showed a median increase of 152 cells/mm^3^ [IQR −4–398] from the time of switch until the end of follow-up, resulting in a median CD4 count of 384 cells/mm^3^ [IQR 204–586] at end of follow-up. The CD4 count change in 27 children was 27 cells/mm^3^ [IQR −172–210] and median CD4 count at end of follow-up was 696 cells/mm^3^ [IQR 357–1189]. At the end of follow-up, immunological failure according to WHO criteria was seen in twenty-four percent of adult patients. Clinical and immunological outcomes are summarized in Table 2.

**Table 2:** Clinical and immunological outcome at end of follow-up and after one year of second-line ART.

Clinical outcome at end of follow-up	ITT, N = 191; N (%)	
In care and on treatment	139 (72.8)	
Transferred out	15 (7.9)	
Lost to follow-up	26 (13.6)	
Death	11 (5.8)	
**Immunological response**	**Adults; median [IQR] or N (%)**	**Children≥5; median [IQR]**
CD4 12 months after start 2nd line	354 [194–508] (OT, N = 91)	641 [459–1245] (OT, N = 21)
CD4 at end of follow-up	384 [204–586] (ITT, N = 152)	696 [375–1189] (ITT, N = 27)
CD4 change from switch to end of follow-up	152 [−4–398] (ITT, N = 152)	27 [−172–210] (ITT, N = 27)
Immunological failure at end of follow-up	37 (24.3) (ITT, N = 152)	

Patients who switched following first-line treatment failure.

ITT: intention-to-treat analysis, OT: on-treatment analysis, N: number of patients, IQR: interquartile range.

Immunological failure was defined according to the WHO guidelines: a CD4 count after six months of therapy below 100 cells/mm3 or below the pre-therapy count, or a 50% decline from the on-treatment peak CD4 count value. [1]

### Virological response on 2^nd^ line ART

**Figure 1:**
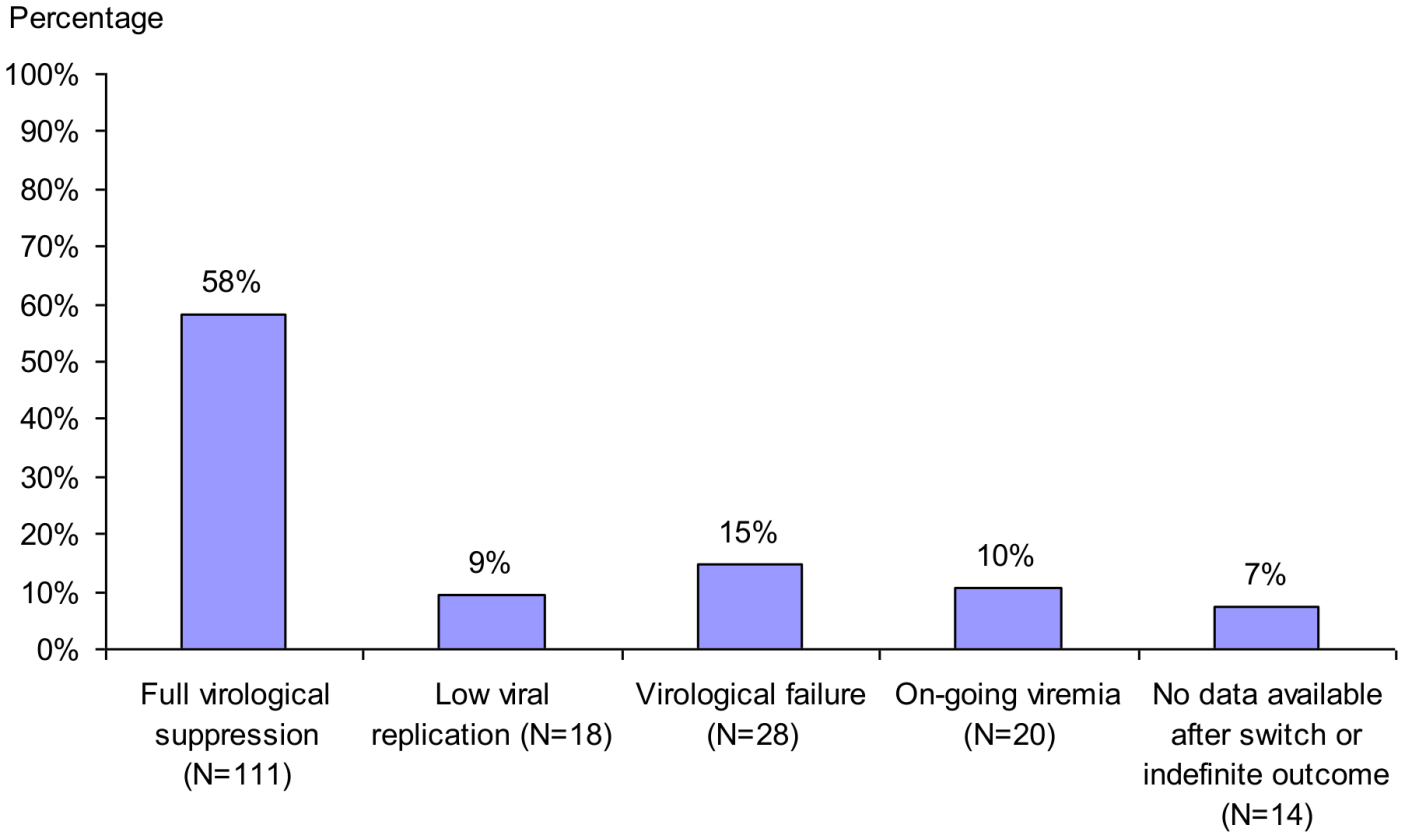
Virological outcome at end of follow-up; N = 191. Patients who switched following first-line treatment failure. N: number of patients, VL: plasma HIV-1 viral load. Full virological suppression: plasma HIV-1 RNA<50 copies/ml, low viral replication: 50<plasma HIV-1 RNA<400 copies/ml, virological failure: plasma HIV-1 RNA>1000 copies/ml after initial VL<400 copies/ml, on-going viremia: plasma HIV-1 RNA never<400 copies/ml. Indefinite outcome: duration of follow-up <6 months without adequate virological response.

**Figure 2:**
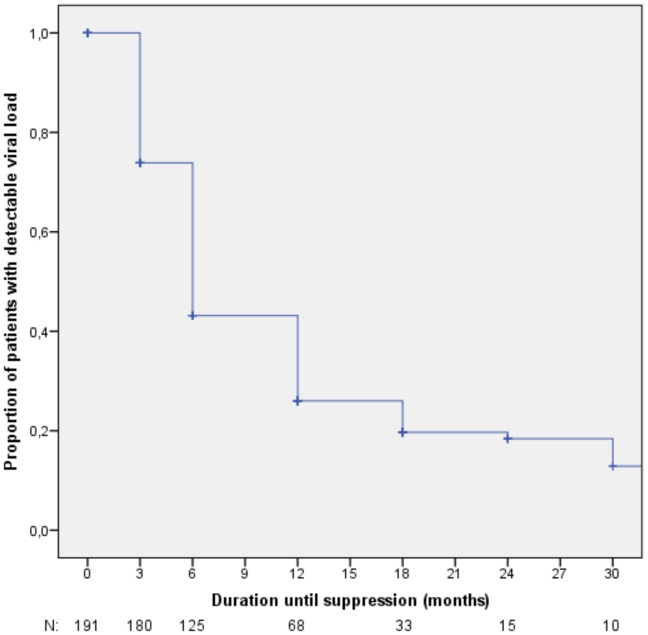
Initial full virological suppression (plasma HIV-1 RNA<50 copies/ml). Patients who switched following first-line treatment failure. N: number of patients at risk.

Cross-sectional analyses showed an adequate virological response (plasma HIV-1 RNA<400 copies/ml) in 71.9% (92/128, OT) of patients after one year, and in 75.0% (63/84, OT) after two years of second-line ART; 60.9% (78/128, OT) and 64.3% (54/84, OT) experienced full virological suppression (plasma HIV-1 RNA<50 copies/ml) after one and two years respectively. Of the 191 patients who had started second-line ART following first-line treatment failure, 67.5% (129/191, ITT) showed an adequate virological response and 58.1% (111/191, ITT) full virological suppression at the end of follow-up. No differences in virological response between age groups were observed. Virological results are summarized in Figure 1 and Table 3. Full virological suppression over the course of time is shown in Figure 2.

**Table 3:** Virological response after start second-line. Cross-sectional analysis, OT.

Virological response after start 2^nd^ line	Total, % (N)	Adults, % (N)	Children, % (N)
**6 months**			
Plasma HIV-1 RNA<400 copies/ml	70.9 (95/134)	70.8 (75/106)	71.4 (20/28)
Plasma HIV-1 RNA<50 copies/ml	55.2 (74/134)	57.7 (61/106)	46.4 (13/28)
**12 months**			
Plasma HIV-1 RNA<400 copies/ml	71.9 (92/128)	70.8 (68/96)	75.0 (24/32)
Plasma HIV-1 RNA<50 copies/ml	60.9 (78/128)	60.4 (58/96)	62.5 (20/32)
**18 months**			
Plasma HIV-1 RNA<400 copies/ml	68.9 (82/119)	68.9 (62/90)	69.0 (20/29)
Plasma HIV-1 RNA<50 copies/ml	58.8 (70/119)	58.9 (53/90)	58.6 (17/29)
**24 months**			
Plasma HIV-1 RNA<400 copies/ml	75.0 (63/84)	76.6 (49/64)	70.0 (14/20)
Plasma HIV-1 RNA<50 copies/ml	64.3 (54/84)	65.6 (42/64)	60.0 (12/20)

Patients who switched following first-line treatment failure.

N: Number of patients.

Treatment failure was seen in 48/191 (25.1%, ITT) patients of which 14.7% (28/191) showed virological failure and 10.5% (20/191) on-going viremia. Consecutive measurements of plasma HIV-1 RNA above 1000 copies/ml were available for almost half (13) of the 28 patients who experienced virological failure; in the remaining 15 patients such a high plasma HIV-1 RNA was only seen once. Median duration of follow-up for the patients with treatment failure was 20 months [IQR 13–30] compared to 23 months [IQR 16–39] for the patients achieving virological success, which was not significantly different (*P* = 0.15). No risk factors were found to be significantly correlated with failure on second-line treatment in univariate analysis. However, an association with the outcome (*P*<0.50) was found for the following risk factors: gender, CD4 count at moment of switch and duration of second-line ART, and were thus included in multivariable analysis. After multivariable analysis none of these factors were found to be independently associated with treatment failure.

We observed a lack of correlation between immunological and virological failure; only 24 of the 39 (61.5%) adult patients experiencing virological failure or on-going viremia showed immunological failure. Reversely, an adequate virological response was seen in 13 of the 37 adults (35.1%) with immunological failure according to the WHO-guidelines.

All analyses were repeated on the group of 210 patients including the 19 patients who had switched to second-line ART for other reasons than first-line treatment failure. These analyses generated similar results for virological, clinical and immunological efficacy (data not shown).

## Discussion

In this cohort in rural South Africa, good virological response on second-line antiretroviral therapy following first-line failure was seen during a long period of follow-up. This observational study reflects the actual efficacy of PI-based second-line ART in our cohort, in which regular virological monitoring is routine clinical practice.

Only few other reports on virological efficacy of PI-based second-line ART in sub-Saharan Africa have been published, most of them from urban areas. One group from Malawi reported on second-line treatment in a prospective observational study, where virological monitoring was performed every three months. Results of this cohort showed higher numbers of virological suppression after twelve months of second-line therapy. [11] Although these results are promising, three-monthly virological testing does not seem to be feasible in RLS where financial restrictions limit the possibilities of frequent monitoring. In an urban South African cohort in which the frequency of virological monitoring was the same as in our cohort, similar proportions of patients reaching virological suppression <400 copies/ml were seen after one year of second-line ART in on-treatment analyses. Results of intention-to-treat analyses were not reported in this study. [12] Two other studies from South Africa showed comparable results, although they only reported short-term (6-month) efficacy of second-line therapy. [13], [14] A recent pilot study conducted in diverse RLS investigated the efficacy of lopinavir/ritonavir monotherapy as a second-line regimen. This strategy resulted in a high proportion of patients reaching a plasma HIV-1 viral load below 400 copies/ml after half a year, which is a promising result. However, the long-term efficacy in clinical practice of this strategy remains to be seen. [22]

In most of the ART efficacy studies in RLS, a cut-off point of plasma HIV-1 RNA<400 copies/ml was used to define an adequate virological response. However, in developed countries, a more strict value (plasma HIV-1 RNA<50 copies/ml) is generally used to define virological suppression. [23], [24] Low-level viremia, with plasma HIV-1 viral loads remaining between 50 and 400 copies/ml, may indicate on-going viral replication and could therefore lead to the selection of drug-resistance mutations and subsequent virological failure. [25] In the presence of a boosted PI the clinical relevance of low-level viremia may be limited. Longer follow-up is needed to find out how many patients with such low-level viremia will eventually indeed experience virological failure. Although the number of patients reaching a plasma HIV-1 viral load<400 copies/ml in our cohort seems adequate and remained stable after 12 and 24 months, the number of patients experiencing full virological suppression (plasma HIV-1 viral load<50 copies/ml) was clearly lower (60.9% versus 71.9% and 64.3% versus 75.0% respectively).

Virological efficacy of second-line PI-based ART in children in the current study was similar to adult patients. This finding was remarkable, considering the first-line (NNRTI-based) regimen outcomes in this cohort, where treatment failure occurred more often in children than in adults. These analyses were performed amongst patient groups that were comparable to the subjects included in the current study. [20], [21] The relatively good outcome amongst pediatric patients suggests that a PI-based regimen in children results in a better virological response compared to an NNRTI-based regimen. A recently published trial on HIV-treatment in therapy-naïve children in RLS indeed reports a boosted PI to be superior over nevirapine in first-line ART. [26]

Treatment failure (virological failure or on-going viremia) had occurred in a quarter of patients at the end of follow-up, numbers being comparable to the earlier mentioned studies on PI-based second-line ART in sub-Saharan Africa. [11]–[14] Higher proportions of patients (35–40%) experiencing failure on second-line therapy, based on virological parameters, were found in two other, South African studies. However, these studies had a cross-sectional design and the duration of second-line ART was not mentioned for all patients in those studies. It was not mentioned if routinely virological monitoring was performed at the study sites. [15], [16]

Mortality within twelve months after treatment switch and at the end of follow-up was low, in contrast to the previously described high early mortality rates after initiation of first-line ART in RLS. [5], [27] On the other hand, the numbers are comparable to those observed by other South African studies on the efficacy of second-line ART in settings with virological monitoring. [13], [14] In a study that used clinical and immunological parameters to define treatment failure, however, a higher mortality rate was observed (10% mortality within the first year after treatment switch). [6] A mortality rate of 5.4% was seen in an African and Asian multicohort study, in which mainly clinical parameters were used as well. [28]

The median CD4 count increase from start of second-line ART until end of follow-up among adults was substantial and similar to that reported by other observational studies. [11], [12]

In this study, the correlation between immunological and virological failure on second-line ART seemed to be minimal. As virological failure precedes immunological and clinical failure, patients experiencing treatment failure were probably frequently detected prior to overt CD4 count decreases and clinical deterioration had occurred, because of regular virological monitoring. [29]–[32] Alternatively, an adequate virological response was seen in more than one third of patients showing WHO-defined immunological failure. This might prevent unnecessary treatment switches with increased costs in the future when consecutive regimens will become available. Such viro-immunological dissociation was previously observed in our cohort at the moment of first-line treatment failure, and has been described in other reports as well. [5], [21] These findings emphasize the need for virological monitoring in patients on antiretroviral therapy as a way to detect treatment failure.

There are some limitations to this study. First, as it is a retrospective observational study, there may be unmeasured underlying determinants influencing results. Second, there were some missing data, mainly due to patients being lost to follow-up. Unfortunately, causes of attrition in these patients were unknown. Third, adherence was not systematically measured in our cohort. Further, analyses on immunological response were only possible for adult patients and children of 5 years and older, as CD4 percentages were not available for the small group of pediatric patients under the age of 5. Last, there was a considerable diversity in time of follow-up after start of second-line treatment, limiting the number of patients with prolonged follow-up times (>18 months).

In summary, this observational cohort study shows that second-line antiretroviral therapy in a rural area in South Africa can result in an adequate and sustained virological response in a significant number of patients, in both adults and children, following first-line treatment failure. Unfortunately, full virological suppression is seen in only about sixty percent of patients. As a result, persisting low-level viral replication may occur in some patients. It remains to be seen whether this is of clinical relevance in PI-based regimens. Regular virological monitoring is necessary to detect treatment failure before immunological deterioration occurs.
